# Factors Influencing the Mental Health of First-Year College Students: Evidence from Digital Records of Daily Behaviors

**DOI:** 10.3390/bs15050618

**Published:** 2025-05-02

**Authors:** Jingyi Ou, Kunyu Wang, Mingzhang Zuo, Di Chen, Heng Luo

**Affiliations:** Faculty of Artificial Intelligence in Education, Central China Normal University, Wuhan 430079, China; oujingyi@mails.ccnu.edu.cn (J.O.); ccnuwky2019@mails.ccnu.edu.cn (K.W.); mzzuo@mail.ccnu.edu.cn (M.Z.)

**Keywords:** mental health, influencing factors, daily behaviors, first-year college students, self-management

## Abstract

The mental health of college students, particularly first-year students, deserves significant attention. As they transition from high school to college, they experience substantial changes not only in their learning behaviors but also in their daily behaviors, which may pose mental health challenges. However, existing studies exploring the factors influencing college students’ mental health still have limitations in the selection of predictor variables and behavior assessment. Building on this, our study adopted a cross-sectional design involving 110 first-year college students in China (M = 17.94 years; 47 males, 63 females) and analyzed the impact of daily behaviors on mental health. We collected daily behavioral data through a designed mini program, and assessed mental health using the GHQ-28. LASSO regression was applied to identify key behavioral predictors, and semi-structured interviews were conducted to further explore the impact of these factors. The results show that sleep quality, planned and implemented physical exercise, average self-evaluation scores, average daily number of planned tasks, and the completion rate of planned tasks have a positive impact on mental health. These findings contribute to enhancing the theoretical understanding of how daily behaviors shape college students’ mental health and offer practical guidance for universities to carry out mental health education.

## 1. Introduction

Rapid societal development and transformation has caused college students to face unprecedented pressures and challenges. As a result, their mental health issues are increasingly prominent, becoming a focal point of widespread attention in both academic circles and society (Torres et al., 2017). Currently, college students not only have to cope with heavy academic loads but also face various pressures, such as intricate interpersonal entanglements and future uncertainty. These pressures often lead to emotional distress, which may manifest as anxiety and depression (Beiter et al., 2015), severely affecting their learning and life. A survey study in the United States showed that the proportion of college students diagnosed with mental health issues on campus had risen from 21.9% to 35.5% over the past decade (Lipson et al., 2019). A 2018 survey conducted by the World Health Organization estimated that over 30% of college students from eight countries had experienced mental distress (Auerbach et al., 2018). The mental health status of college students in China also appears to be deteriorating. For example, the recent study of Cheng et al. (2021) revealed that the prevalence of depressive symptoms among college students stands at 31.2%, surpassing the rate observed in the general Chinese population. These survey results suggest that the mental health of college students is an important social issue that should not be ignored.

Among college students, the mental health issues of first-year students are particularly prominent. They are experiencing the entry into adulthood from adolescence while facing a brand-new environment and complex challenges away from home, and need to independently handle various issues such as academic studies, life, and interpersonal relationships (Tosevski et al., 2010). Environmental changes often accompany changes in a person’s psychological state. Research has shown that life transitions are often accompanied by increased depression, anxiety, and stress (Beiter et al., 2015; J. Wang et al., 2019). If these issues are not properly resolved, they may lead to serious consequences such as poor academic performance and an increased risk of dropping out of university (Hjorth et al., 2016); this could not only affect the student’s current education and quality of life but also have long-term negative impacts on their future development. Therefore, correctly recognizing and addressing the mental health issues of first-year students is an important issue facing universities and society (Lei et al., 2021).

The mental health of college students is shaped by a complex interplay of behavioral and structural factors (Campbell et al., 2022), including physical activities, mental activities, communication styles, interaction patterns, and daily lifestyles (Yosep et al., 2024). Among these factors, simple daily behaviors under an individual’s control have emerged as particularly important contributors to mental health (Velten et al., 2014). Previous studies have shown that regular sleep patterns (Brooks et al., 2009), good sleep quality (Dagani et al., 2024; A. J. Scott et al., 2021), appropriate exercise (Chekroud et al., 2018), robust social networks (Campbell et al., 2022), and enjoyable leisure activities (Das et al., 2019) may have a positive impact on mental health by reducing stress and anxiety and improving emotional states such as boosting self-esteem. In contrast, excessive reliance on electronic devices may have negative effects, including increased stress, reduced emotional connection, and distraction (D. A. Scott et al., 2017).

Although previous studies have examined the relationship between college students’ daily behaviors and mental health, the methods for collecting behavioral data are often simplistic, typically relying on one-time self-reports and limited predictor variables. These approaches lack continuous monitoring and fail to capture the complexity and situational context of behaviors. Therefore, this study focuses on first-year college students and employs a mini program to collect behavioral data over an extended period, allowing for more consistent and detailed observation. This method provides a more precise understanding of how various behavioral factors relate to mental health.

## 2. Literature Review

### 2.1. Mental Health

#### 2.1.1. Definition of Mental Health

Mental health, a complex yet essential aspect of human well-being, has been broadly conceptualized by various institutions and experts. The World Health Organization characterizes mental health as a state of well-being in which individuals are cognizant of their capabilities, resilient to life’s typical stresses, productive in their work, and able to contribute positively to their communities (World Health Organization, 2004). This definition underscores the individual’s ability to function optimally in societal contexts.

Expanding upon this perspective, in 2006, the Public Health Agency of Canada emphasized the multidimensional nature of mental health, describing it as the capacity to experience emotions, thoughts, and behaviors that enhance our ability to enjoy life and navigate challenges. It reflects a positive emotional and spiritual well-being that values cultural diversity, equity, social justice, interconnectedness, and personal dignity (Public Health Agency of Canada, 2006). Context and cultural sensitivity are emphasized as crucial elements in understanding mental health by this definition.

Building upon these foundational perspectives, the World Health Organization provided a more recent and holistic definition of mental health in 2022. This definition recognizes mental health not merely as the absence of mental disorders, but as “a state of mental well-being that enables people to cope with the stresses of life, realize their abilities, learn well and work well, and contribute to their community” (World Health Organization, 2022, p. 8). Compared with the 2004 definition, the 2022 version adopts more concise language and explicitly emphasizes the notion of “a state of mental well-being”, thereby further clarifying the conceptual core of mental health. Moreover, it places greater emphasis on overall functioning in learning and work, while relatively downplaying the previous focus on “being productive”.

In summary, mental health is a multifaceted state of well-being that enables individuals to recognize and utilize their capabilities, adapt to life’s stresses, achieve personal potential, engage effectively in learning and work, and contribute positively to their communities. Mental health is a fundamental component of overall health, essential to individual growth, relationship-building, and societal advancement (U.S. Department of Health and Human Services, 1999).

#### 2.1.2. The Vulnerable Mental Health Status of First-Year College Students

The mental health of college students, particularly first-year college students, warrants close attention given the significant challenges they encounter during this transitional phase. This concern is supported by many empirical studies showing a high prevalence of mental health issues in this population. For example, Adams et al. (2021) conducted a longitudinal cohort study on 1686 first-year university students. The results showed that about 30% of the students had symptoms of anxiety and depression, and this proportion increased by the end of the academic year. Similarly, a cross-national study found that the prevalence of mental disorders among first-year students was very high, with data from 72,288 students across 18 countries (Mason et al., 2025). This may be due to the transition from high school to university, which encompasses momentous changes in living and learning environments, often imposing considerable pressure on students (Cleary et al., 2011). This pressure is further exacerbated for first-year college students, who must contend with academic demands while addressing the various challenges posed by environmental changes (Tosevski et al., 2010; Verger et al., 2009). These pressures and experiences are not just associated with an increased risk of mental illness; they can also have a detrimental impact on students’ academic performance (Kessler et al., 1995) and future interpersonal relationships (Kessler et al., 1998).

In addition to being at a critical transitional stage, first-year college students are facing psychological challenges that include the subtle yet profound impacts of the COVID-19 pandemic since high school. The pandemic has introduced widespread psychological and social disruptions that have already harmed and will continue to influence the mental health of young people (Meherali et al., 2021). For students who entered college in the past two years, these challenges were compounded by the pandemic’s effects during their high school years. For instance, remote learning during the pandemic increased their reliance on electronic devices, elevating the risk of smartphone addiction—a factor that may heighten vulnerability to mental health issues such as depression (Wu & Liu, 2023). Moreover, home confinement measures significantly reduced opportunities for physical activity (Das et al., 2024), which is widely recognized for its positive impact on mental health and overall well-being (Talapko et al., 2021). Changes in exercise habits caused by the pandemic may therefore have contributed to heightened levels of anxiety, depression, and feelings of loneliness (Amo et al., 2022). Overall, the long-term effects of the pandemic have led to substantial shifts in students’ lifestyles and learning environments (Meherali et al., 2021), exerting profound influences on their psychological well-being.

Consequently, first-year college students are considered a high-risk group for mental health issues. It is imperative that we accord them sufficient attention during this crucial transitional period, providing timely support and assistance to address their mental health concerns.

#### 2.1.3. Mental Health Differences Among Student Demographics

Many studies have revealed that demographic variables have an impact on the mental health status of college students (C. Wang et al., 2022). Among these, gender stands out as an important characteristic that significantly influences mental health. Generally speaking, the mental health status of male students is higher than that of female students, which can be traced back to the pivotal role that societal expectations and gender roles play in shaping mental health outcomes (Bhatia & Goyal, 2020). Males are often socialized to emphasize achievement, competitiveness, and autonomy, while female students are encouraged to prioritize relationships, emotional expression, and caregiving (Betz & O’Connell, 1989). Several empirical studies have confirmed that these differing gender roles can result in unique stressors and coping mechanisms, affecting mental health.

For example, Nogueira et al. (2022) conducted a cross-sectional study on 560 first-year college students in Portugal, collecting their psychological data through Mental Health Inventory (MHI-38) and Psychological Vulnerability Scale (PVS). The results showed that female students scored lower on each subscale of MHI-38 and higher on self-vulnerability. Additionally, in a study examining factors affecting mental health among Chinese college students, H. Li et al. (2008) classified student mental health status into three groups: Group A (severe psychological issues), B (adaptive issues), and C (psychological well-being). They found that gender was a statistically significant factor distinguishing Group A from Group C.

Alongside gender, only-child status is also a noteworthy demographic variable. In the context of China, children with siblings are often reported to exhibit better mental health outcomes compared to only children. This pattern may be explained by several factors. First, only children often face heightened expectations from their parents, leading to increased pressure and anxiety (Fan, 2016). Second, they lack siblings who could provide emotional support and conflict resolution, potentially affecting their social skills and resilience (Fukuya et al., 2021).

P. Chen et al. (2024) utilized the Symptom Checklist 90 (SCL-90) to compare the mental health status of children and adolescents from one-child families and multi-child families in China. The results indicated that children and adolescents from one-child families scored lower in multiple dimensions, including depression and anxiety. Luo et al. (2021) reached a similar conclusion through a systematic review and meta-analysis of the prevalence of depressive symptoms among Chinese college students. Expanding upon this conclusion, Fan (2016) found that while the mental health status of non-only children is significantly better than that of only children to a certain extent, there is a trend of mental health indicators first improving and then declining as the number of siblings increases. This emphasizes the need to analyze the mental health differences caused by the only-child status specifically and circumstantially, rather than by applying generalized conclusions.

In terms of geographical location, students from urban areas tend to exhibit superior mental health compared to their rural counterparts. This may be due to differences in the availability of mental health resources and services, as well as varying degrees of environmental changes. For example, urban locality often provides easier access to mental health resources and services, including a more diverse range of mental health experts and treatment options (Zhang et al., 2017). The accessibility of these resources helps in earlier identification and intervention for mental health issues, leading to better outcomes for urban students. Further, the development of rural students differs from their urban counterparts as a result of historical and economic factors. These disparities may pose greater challenges for rural students adapting to life and environmental changes in urban settings, where most universities are found. As they adapt, rural students may be more prone to psychological issues due to their reliance on familiar family and cultural values, which may be absent or altered in an urban environment (Zhang et al., 2017).

There is some evidence supporting the aforementioned theory; Tao et al. (2002) adopted the Self-Rating Anxiety Scale (SAS) and Self-Rating Depression Scale (SDS) to assess the mental health status of 1134 college students and explore the impact of demographic variables, including the place a student was raised in, on mental health. The results revealed that students raised in cities scored lower in depression and anxiety than those brought up in towns or rural areas. Similarly, measurements of the mental health status of rural and urban university students using four standardized mental health scales showed that urban students scored significantly higher than rural students in terms of self-esteem and social support (Zhang et al., 2017).

### 2.2. Potential Impact of Daily Behaviors on Mental Health

In addition to demographic differences, daily behaviors also have the potential to impact the mental health of college students (Tao et al., 2002). Some studies have indicated that depression among college students can be largely attributed to their daily behaviors in life (Liu et al., 2022). The following sections describe several commonly mentioned behavioral factors and corresponding evidence-based studies:

First, numerous studies have confirmed the significant impact of sleep on mental health. For example, A. J. Scott et al. (2021) conducted a meta-analysis of 65 randomized controlled trials reporting on the impact of sleep improvement interventions on overall mental health. The analysis revealed that improving sleep has a significant moderate effect on overall mental health, indicating that an improvement in sleep quality leads to an improvement in mental health. Additionally, correlation research on insomnia symptoms and mental health problems among 373 college students using questionnaire survey and sleep diary methods showed that, compared with college students without insomnia symptoms, those with insomnia symptoms had high scores and poor mental health status in somatization, obsessive-compulsive, depression, anxiety, and global severity indices (D. J. Taylor et al., 2011). Supporting this evidence, a recent study of over 700 Italian first-year college students found that dissatisfaction with sleep quality was significantly associated with elevated psychological distress. It is worth noting that the study examined both sleep duration and sleep quality, with the latter emerging as the more critical factor (Dagani et al., 2024).

Second, exercise also has a positive impact on mental health by alleviating anxiety and improving mood (C. B. Taylor et al., 1985), thereby also improving mental health status (Chekroud et al., 2018). For example, a study conducted by Grasdalsmoen et al. (2020), utilizing data from the Norwegian Student Health Survey (SHoT2018), analyzed the relationship between exercise habits and mental health issues among 50,054 university students. They found that exercise frequency, intensity, and duration were inversely correlated with measures of mental health issues, with exercise frequency showing the most significant association. Another large-scale longitudinal study involving 164,101 students from 22 Chinese universities reported similar conclusions, emphasizing the negative impact of lack of exercise on mental health (Y. Li et al., 2021).

Third, reading plays a significant role in regulating individual emotions and maintaining mental health. In 2017, Merga (2017) conducted a survey among 1022 adult readers, analyzing their open-ended responses. The study found that people often use reading as a coping mechanism to escape real-life problems. The relaxation brought by reading provides effective support for mental health as an outlet for emotional experiences and expression. Japanese scholar Miyata studied 881 Japanese individuals using a cross-sectional design to examine how daily reading habits, including duration and speed, relate to psychological states. There was a positive correlation between reading duration and increased well-being, positive emotions, and empathy, and a negative correlation with depression (Miyata, 2020).

Fourth, the way individuals spend their daily leisure time (e.g., hobbies, interests, and socializing) influences their physical and mental well-being (Toyoshima et al., 2016); leisure enhances well-being while alleviating stress and depression (Fullagar, 2008; Harvey et al., 2010). Das et al. (2019) conducted a descriptive survey of 310 Indian university students using two tailored questionnaires, discovering that participation in social activities resulted in better mental health. The study also revealed a significant impact of leisure time on mental health. This was confirmed by another cross-sectional study that explored the individual and interpersonal factors affecting the mental health of 2203 public university students, which found a positive correlation between social participation and improved mental health (Byrd & McKinney, 2012).

Lastly, the excessive use of electronics may negatively impact the mental health of college students. Research suggests a correlation between depressive emotions and internet usage (Elhai et al., 2018; Huckins et al., 2019). One literature review assessed Chinese college students’ smartphone usage and mental health status, and collected data such as the time spent on smartphones via online questionnaires. The results showed that long-term smartphone usage had a negative impact on the mental health of college students (Z. Wang & Zheng, 2020).

### 2.3. Research Questions

However, it must be noted that existing studies on the impact of daily behaviors on college students’ mental health have limitations. First, the methods used to collect behavioral data are overly simplistic, primarily relying on one-time self-reports such as traditional questionnaires (Norbury & Evans, 2019; Tyson et al., 2010), which lack continuous monitoring of behavior over a period of time. This limits the accuracy and reliability of the data obtained. Second, the selection of predictor variables in existing studies is often limited, as they are often limited to a single variable and fail to comprehensively consider the overall picture of learning and living habits. Moreover, behavior assessment often focuses only on overt characteristics, such as frequency and duration (Chekroud et al., 2018; Grasdalsmoen et al., 2020; Z. Wang & Zheng, 2020). It overlooks other important aspects, like the specific situational conditions under which behaviors occur and individuals’ self-evaluations of their behaviors. To better understand the complexity and dynamics of behaviors and the impact of daily behaviors on mental health, we need to further explore the detailed elements of behaviors and improve the methods of assessment.

To deepen the exploration of this field and further enrich the relevant research, this study investigates the daily behavioral factors that affect the mental health of first-year college students by adopting a designed mini program to record long-term and detailed daily behavioral data. This digital recording is designed to comprehensively collect process-oriented behavioral data from students within a certain period, rather than simply relying on questionnaires. We then used the LASSO regression data mining methods to screen out the key factors that affect the mental health of first-year college students. Supplementary semi-structured interviews were conducted to further explore how these factors specifically affect mental health. This study aims to provide targeted suggestions for the mental health development of first-year college students, helping them better cope with challenges and achieve personal development. The main research questions discussed in this article are as follows:

1. What is the overall mental health status of first-year college students in China?
2. How does mental health status differ among student demographics?
3. Which daily behaviors significantly predict student mental health status?

## 3. Methods

### 3.1. Ethics Statement

The research study was reviewed and approved by the Institutional Review Board of Central China Normal University. All methods were performed in strict accordance with relevant guidelines and regulations. Written informed consent forms were obtained from all participants before the study. All participants were made aware that their participation in the study was voluntary, and their personal identifiable information would be kept anonymous at all publications and presentations. Participants had the right to choose not to participate and could withdraw at any time without penalty or adverse effects. To ensure confidentiality and minimize observation-related bias, all data were anonymized by a teaching assistant before analysis, so that instructors could not link responses to individual participants. Participants were also informed of this arrangement to reduce concerns and promote honest reporting.

### 3.2. Participants

This study was conducted over the 2023 fall semester, mainly relying on a first-year seminar course offered at a research university in central China. On 26 September 2023, during the first lecture of the course, we explained the purpose and protocol of the study to the students and distributed informed consent forms for them to sign, indicating their willingness to participate. The recruitment period concluded on 30 September 2023. A total of 110 first-year undergraduate students (47 males and 63 females) voluntarily participated in the research. They were between the ages of 17 and 20 (M = 17.94, standard deviation (SD) = 0.64). The detailed demographic information of the participants is shown in Table 1. Demographic information such as ethnicity and socioeconomic status was not included in this study. The majority of participants were Han Chinese, limiting the feasibility of meaningful ethnic comparisons. Additionally, socioeconomic status was considered sensitive, but students’ place of upbringing was collected as a proxy indicator.

Although the sample size may be somewhat limited in breadth, unable to cover a wider range of individuals, we took into consideration that the data collection was conducted through continuous digital records rather than traditional questionnaires. This method inherently restricts large-scale sample collection. Therefore, we chose to compensate for the lack of breadth by focusing on the depth of the study. By conducting long-term and detailed follow-up records of these samples, we aimed to deeply explore the relevant data and phenomena, thereby ensuring the reliability and validity of the research findings.

### 3.3. Research Design and Procedure

This study employed a cross-sectional design to investigate the daily behavioral factors that influence the mental health status of first-year college students. Cross-sectional design is a social science research method primarily used to study the conditions or characteristics of a group of individuals, populations, organizations, or society at a specific point in time. The core feature of this method is its cross-sectional nature, which involves conducting a one-time survey and the observation of individuals within a group or social phenomenon to gather information about that group (X. Wang & Cheng, 2020). The specific research process is as follows:

This research centered on a first-year student seminar that aimed to assist first-year students in making the transition from high school to university. A core component of the course was the completion of a four-week WeChat mini program recording activity. WeChat is a widely used social media and messaging app in China, with over one billion monthly active users (WeChat, 2025). Participants were required to record their daily behaviors of learning and living as much as possible, in order to better plan, monitor, reflect on, and adjust their behaviors accordingly.

First, we collected daily behavioral data in digital records through the mini program. To eliminate the novelty effect and reduce errors, students were familiarized with the program over a one-month preliminary data collection session prior to the official start of the recording activity. Preliminary data were reviewed and organized to gain insights into the various issues encountered by students during the use of the mini program. These findings were used to provide guidance, help students familiarize themselves with the mini program’s operation methods, record procedures, and understand project goals. This ensured that during the formal data collection process, data would be reported accurately and comprehensively.

Afterward, we officially launched the WeChat mini program recording activity. Over four weeks, students self-reported detailed behavioral data daily on the mini program. Students were sent nightly (10 p.m.) reminders through an online group chat tool to ensure the completion of the daily record.

After the recording activity ended, we distributed and collected a mental health questionnaire (General Health Questionnaire, GHQ-28) to score the participants’ recent mental health status. Based on the questionnaire data, we randomly selected students from groups with higher, average, and lower scores and invited them to participate in interviews. This measure aimed to gather complete perspectives from individuals with different mental health scores, thereby obtaining more objective and comprehensive information.

Finally, we conducted face-to-face semi-structured interviews, each lasting approximately 20–30 min. The interviews were recorded with the consent of the participants. During the interviews, we asked participants to view a visual presentation of their one-month digital records and encouraged them to share their experiences and opinions. Through these interviews, we collected qualitative data to further understand their perspectives on factors affecting mental health, which provided subjective interpretations of the statistical results.

### 3.4. Instruments

This study collected both quantitative and qualitative data. The quantitative data included daily behavioral data and mental health scores, while the qualitative data consisted of interview data. The tools used for each type of data are described below:

#### 3.4.1. Digital Records of Daily Behaviors

**Figure 1:**
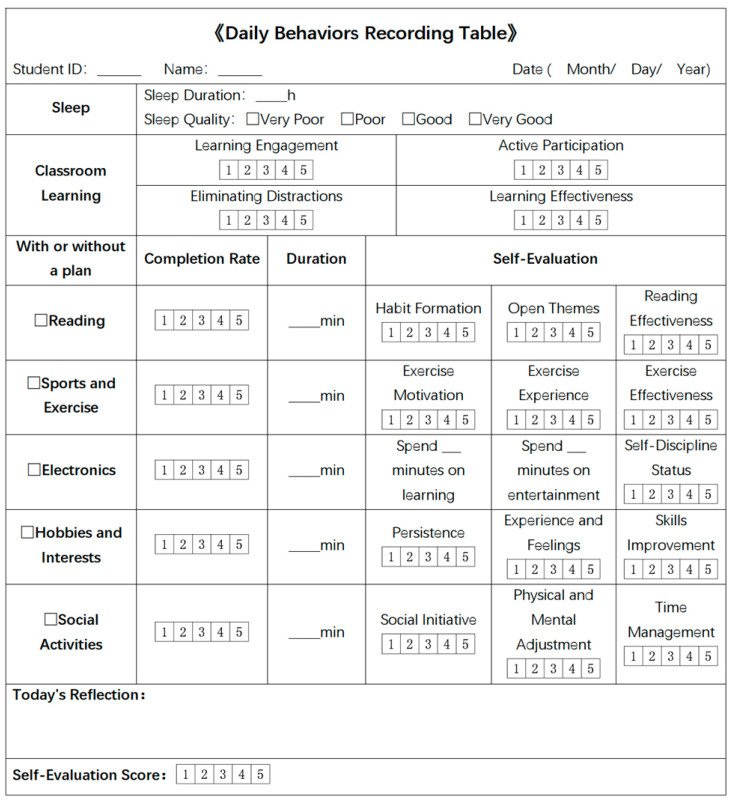
Daily behaviors recording table.

**Figure 2:**
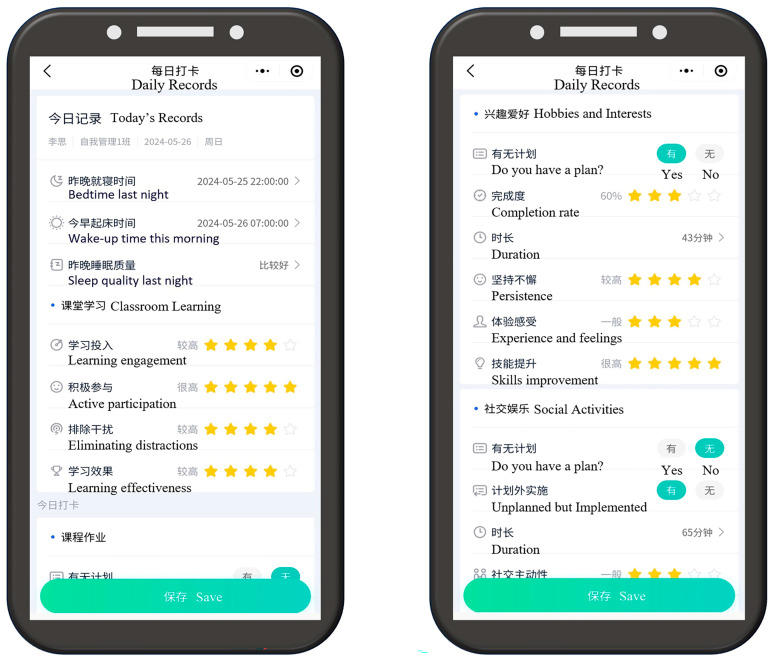
Interface for using the mini program.

We first categorized behavior types into various sections, with each section encompassing detailed information on specific behaviors. Based on this categorization, we produced a daily behavior recording table (Figure 1). Utilizing this table as a foundation, we then designed a digital recording tool, a WeChat mini program (Figure 2). The fixed content requirement of the mini program ensured data standardization and reporting consistency. The recorded variables were selected based on previous research and covered multiple daily behavioral factors including sleep, classroom learning, reading, physical exercise, electronic device usage, hobbies and interests, social activities, daily reflections, and self-evaluation scores. We evaluated multiple elements of each behavior, including planning, completion, duration, and self-evaluation, to create a comprehensive analysis of daily behavioral patterns.

#### 3.4.2. Mental Health Questionnaire

This study employs the GHQ-28 developed by Goldberg and Hillier (1979) as a tool for assessing participant mental health. Since its creation, the GHQ-28 has been translated into 38 languages and is recognized as one of the most reliable screening tools for evaluating mental health status (Roy & Muhammad, 2021). The questionnaire consists of four dimensions: somatic symptoms, anxiety/insomnia, social dysfunction, and severe depression. It comprises 28 items in total, 7 positively worded and 21 negatively worded. The original scoring mechanism adopts a four-point Likert scale, with higher scores indicating poorer mental health outcomes. Given our specific application and goals, we have made adaptive modifications to the GHQ-28 by adopting a five-point Likert scale to allow for more nuanced responses and accurate expression of feelings. Additionally, to facilitate subsequent data analysis and provide a more intuitive representation of participants’ mental health status, we have reversed the scoring logic so that higher scores now indicate better mental health. This adjustment is more intuitive for most participants and researchers, allowing more rapid understanding of mental health status. The GHQ-28 has been previously validated for measuring the mental health of Chinese college students (C. Chen et al., 2010). Therefore, it is appropriate to adopt this questionnaire as a tool for assessing the mental health of participants in this study. The content of each item is presented in Supplementary Table S1.

To evaluate the reliability and validity of the questionnaire, we examined internal consistency and confirmatory factor analysis (CFA) indices. The overall scale exhibited excellent internal consistency (Cronbach’s alpha = 0.94). As shown in Table 2, the subscales’ reliability and construct validity were supported by their Cronbach’s alpha, average variance extracted (AVE), and composite reliability (CR) values. Although a few AVE values were slightly below the recommended threshold, the corresponding CR values remained acceptable, indicating adequate convergent validity (Fornell & Larcker, 1981). Given the robustness of the original GHQ-28 scale and the minimal modifications made, the overall reliability and validity of the adapted instrument are well supported.

#### 3.4.3. Interview

We conducted an exploratory semi-structured interview to understand the impact of daily behavioral habits on participants’ mental health. The semi-structured format aimed to allow participants to describe and analyze the daily behaviors that influence their mental health. Interviews were guided by the following three questions: (1) Based on the visual results from the mini program, could you please discuss which daily behaviors you believe may have an impact on your psychological state? (2) What changes have occurred in these daily behaviors during the process of digital recording? (3) How do you think these daily behaviors affect mental health? The interviews were conducted according to a preset outline with sufficient flexibility to collect open-ended responses and allow participants’ emotions, perceptions, and suggestions to naturally emerge.

### 3.5. Data Collection

The WeChat mini program digitally gathered the participants’ self-reported daily behavioral data. After the four-week recording activity, we exported all daily behavioral data from the mini program and encoded, integrated, and categorized the data to extract 47 key behavioral variables (Table 3). These variables reflect important behavioral patterns of students in their daily lives including sleep, reading, physical exercise, and the use of electronic devices. We processed the individual-level data using Excel software (version 2021). By merging similar items, calculating frequencies, averages, and other metrics, we obtained numerical values for each participant across all 47 variables.

We collected 110 mental health questionnaires and based on the results, selected individuals from three groups (high, average, and low mental health scores) for semi-structured interviews. Ultimately, we conducted semi-structured interviews with 19 students (lower scores *n* = 6, average scores *n* = 6, higher scores *n* = 7). The interviews explored their perceptions of which daily behaviors affected their mental health, the changes in these behaviors during the recording process, and how these impacts arose.

### 3.6. Data Analysis

#### 3.6.1. Demographic and Mental Health Data

In this study, a descriptive statistical analysis was conducted using IBM SPSS software (version 22) and the significance of differences was determined by means. Descriptive statistical analysis was conducted on the mental health scores. One-way analysis of variance (ANOVA) was used to determine whether there were significant differences in mental health scores across different demographic variables, including gender, place of upbringing, and only-child status. Since the mental health data generally follow a normal distribution, and the variables within each group are independent and exhibit homogeneity of variance, the prerequisites for ANOVA were met.

#### 3.6.2. Daily Behavioral Data

To reduce the risk of missing data, multiple preventive measures were implemented. The mini program included a built-in reminder and validation system that prevented submission if any field was left blank. Additionally, a teaching assistant sent daily reminders through the course group chat, and the system allowed participants to retroactively fill in the previous day’s data.

Across the 110 participants, 60 students (54.5%) completed behavioral records for all 28 days. The average number of recorded days was 25.92 (SD = 4.55), with a median of 28, indicating high overall consistency in daily reporting. On recorded days, all behavioral fields were fully completed, and there were no missing responses in the GHQ questionnaire.

To assess potential bias due to missingness, we compared participants with complete and incomplete records across all 47 behavioral variables and found no statistically significant differences for the majority of them. Given the relatively low proportion of missing data and the limited sample size, participant-level mean imputation was applied to maximize data completeness while maintaining analytical robustness.

Since the daily behavioral data involve both continuous and discrete cases, the possibility a normal distribution was low, and there was a high likelihood of collinearity issues among variables belonging to the same behavior. Therefore, we used the LASSO regression algorithm to screen the key variables for modeling the influence of behaviors on mental health.

LASSO regression, also known as L1 regularization for linear regression, adjusts the complexity of the regression model by introducing a penalty constraint λ (lambda) to the least squares estimation, thereby avoiding overfitting and selecting a smaller set of feature variables. LASSO regression is suitable for situations with skewed sample distributions, effectively eliminating multicollinearity issues among predictors, and it demonstrates excellent performance in compressing feature variables. Compared to the linear regression model, LASSO is better equipped to handle the diverse range and interconnectedness of behavioral variables in this study while also avoiding model overfitting. Therefore, LASSO regression was used to screen factors influencing mental health.

The 47 daily behavioral factors extracted from the check-in records served as the predictors (independent variables) in the LASSO regression model, while the students’ mental health scores constituted the dependent variable (outcome variable). The modeling and computation of the LASSO regression were implemented by calling the glmnet function package in the R programming language.

#### 3.6.3. Qualitative Data

To analyze the interview data, we adopted a grounded theory approach consisting of open coding, axial coding, and selective coding (Strauss & Corbin, 1998). In this study, we conducted the first two stages—open and axial coding—to systematically identify and categorize key themes in students’ narratives. Selective coding was not performed, as our goal was not to construct a full theoretical model, but rather to extract structured qualitative insights that could support and contextualize our quantitative findings.

## 4. Results

### 4.1. Quantitative Results

#### 4.1.1. Descriptive Statistics

Due to the numerous variables recorded by the mini program for daily behaviors, the corresponding descriptive statistics are presented separately in Supplementary Table S2. Descriptive statistics of the questionnaire items collected from 110 total questionnaires are summarized below.

Both the average score per item of the overall scale and the average score per item of each sub-dimension exceeded the benchmark of three points, indicating a moderately high level of scores (Table 4). This suggests that the overall mental health status of the sample population was generally good. Compounding this evidence, for the majority of negative association questionnaire items, the proportion of people choosing “Disagree” and “Strongly Disagree” (and for positive association items, the choices would be “Agree” and “Strongly Agree”) exceeded 50%, indicating that most students exhibited fewer negative mental health signs (Supplementary Table S1).

However, when comparing the scores of the four subscales, the participants scored relatively lower on the “somatic symptoms” dimension, suggesting that despite their good overall mental health, there were some somatic problems. In contrast, participants exhibited higher mental health scores on the “severe depression” dimension, suggesting that they did not show obvious depressive symptoms.

#### 4.1.2. Comparative Analysis

Next, we analyzed whether there were significant differences in mental health scores across gender and sibling status. Interestingly, neither factor produced statistically significant differences in mental health scores among first-year students. Similarly, there were no significant differences in questionnaire scores for first-year college students with different places of upbringing (Table 5).

#### 4.1.3. LASSO Regression

To ensure the statistical validity of the modeling analysis, we first conducted a one-sample Kolmogorov–Smirnov (K–S) goodness-of-fit test on the participants’ daily behavioral data. Consistent with our expectations, the behavioral variables exhibited a non-normal distribution (Supplementary Table S2; for all K–S values, *p* = 0.000). Additionally, we conducted a multicollinearity analysis and confirmed the presence of multicollinearity among the behavioral variables (Supplementary Table S3; multiple variables have VIF values greater than 10), including a correlation exceeding 0.7 between “Planned and Implemented Physical Exercise” and “Frequency of Recording Physical Exercise” (Supplementary Table S4). Given these data characteristics, we used the LASSO regression model to explore and identify the key behavioral characteristics influencing mental health status in the student population.

**Figure 3:**
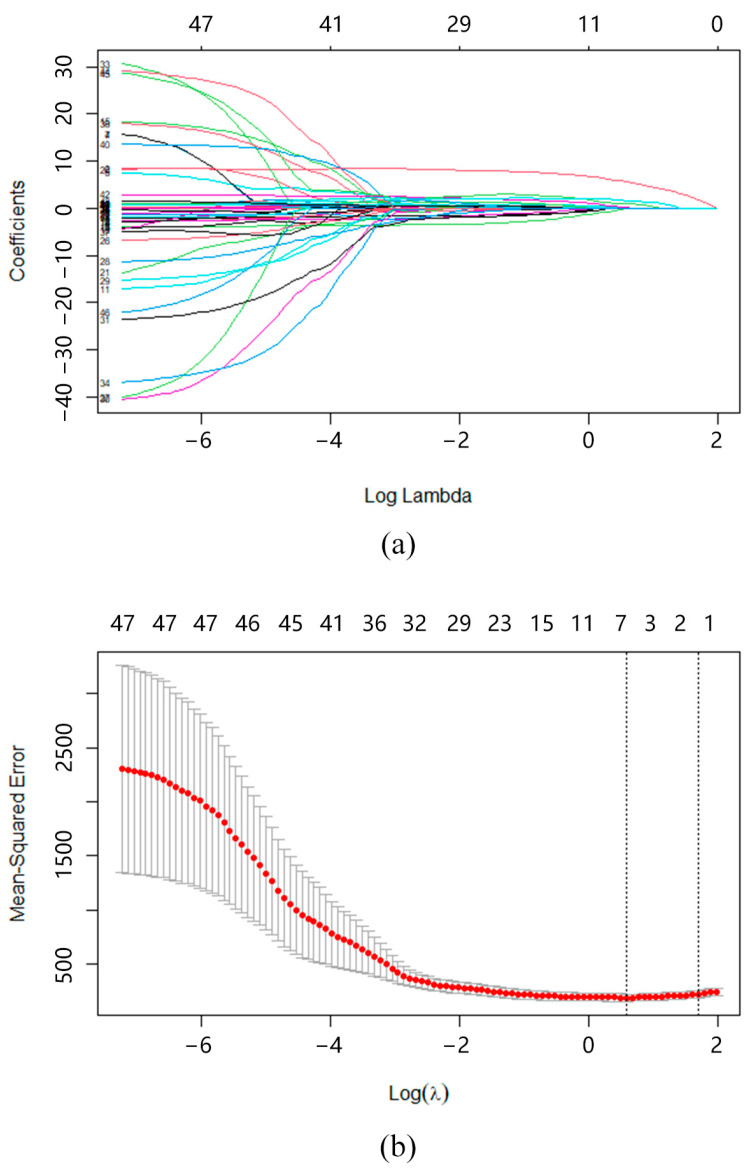
Results of LASSO regression performed on key behavioral determinants of mental health. (**a**) Changes in regression coefficients. (**b**) Changes in mean squared error.

A total of 47 independent variables extracted from the check-in data were included in the LASSO regression model. To determine the optimal value of the regularization parameter λ, we performed 10-fold cross-validation. As the penalty coefficient λ increased, the regression coefficients of the 47 independent variables converged to zero (Figure 3). Simultaneously, the mean squared error (MSE) of the model gradually decreased, reaching its optimal point when logλ was slightly > 0. The optimal λ value, selected based on the minimum mean cross-validated error, was found to be 1.7914. This value was used to fit the final LASSO regression model. At this point, five independent variables possessed non-zero regression coefficients in the LASSO regression model: “Sleep Quality”, “Planned and Implemented Physical Exercise”, “Average of Self-Evaluation Scores”, “Average Daily Number of Planned Tasks”, and “Completion Rate of Planned Tasks”. The five key behavioral variables explained 27.94% of the variance in mental health scores (model determination coefficient %dev = 0.2794).

A variance inflation factor (VIF) check was conducted to assess whether residual multicollinearity persists among selected predictors. It was found that the VIF values for the five retained variables were all below five, indicating that multicollinearity was not substantial enough to bias the regression estimates. These results suggest that the predictive validity and stability of the final model remain reasonably robust. Detailed VIF values are provided in Supplementary Table S5.

While questionnaire-derived gross scores reflected the general quality of students’ mental health, the scores of each sub-dimension give insights into students’ mental health status from different perspectives. Therefore, we conducted further modeling analysis by treating the four sub-dimensions of mental health as dependent variables. We aimed to compare the overall predictive ability of the models for different mental health dimensions (measured by the determination coefficient, %dev) and the importance of the independent variables with non-zero regression coefficients in the models (measured by the standardized regression coefficient, β).

After standardizing the regression and determination coefficients of the five LASSO regression models, we discovered that sleep quality was not only a core factor determining overall mental health status, but that it was also an important factor affecting student performance in the dimensions of “somatic symptoms”, “anxiety and insomnia”, “social dysfunction”, and “severe depression” (Table 6).

### 4.2. Qualitative Results

Quantitative analysis identified daily behavior variables affecting student mental health scores, but an exploration into underlying reasons required further qualitative analysis. To this end, interview audio recordings and transcripts were critically evaluated. During open coding, we identified recurring behavioral and emotional patterns, such as “sleep quality affecting mood” and “exercise improving emotional state”. Through axial coding, these initial codes were organized into five core categories: emotional triggers (e.g., sleep, academic pressure), emotional regulation strategies (e.g., exercise, journaling), task performance and self-evaluation, momentary emotional states, and mental health outcomes. The following findings illustrate how these categories were reflected in students’ narratives, beginning with the most frequently mentioned theme—sleep.

Interviewees generally agreed that sleep significantly impacted their mental health, especially noting that the sleep quality directly affected their emotions. Nine interviewees specifically discussed sleep. They expressed that poor sleep quality could make them prone to “irritability” and “anger”. Furthermore, poor sleep quality also indirectly affects mental health by influencing physical states, such as by causing headaches and other issues. These viewpoints underscore the crucial role of sleep in maintaining individual emotional stability and psychological well-being.

Physical exercise was also considered to have a positive impact on mental health, particularly in relieving emotional stress. Some interviewees pointed out the direct impact of exercise on emotions, stating that exercise can relax tense nerves, bring about a sense of joy, and effectively relieve stress, while others cited an indirect benefit of physical exercise having a positive effect on mental health by improving sleep quality. For example, Interviewee 2 stated, “I find that after exercising, I sleep better and consequently, my mood improves as well”.

Finally, interviewees expressed that having clear plans and a high completion rate of planned tasks significantly improved their mental health. Multiple interviewees indicated that planning helped them reduce the feelings of confusion, alleviate anxiety, and ultimately improve their mental health. Some interviewees expressed that completing tasks brought joy, while failure to complete planned tasks led to feelings of anxiety and unease. A high task completion rate may enhance student satisfaction and mental health, while a low completion rate may lead to negative emotions such as anxiety and unease.

## 5. Discussion

### 5.1. RQ1: What Is the Overall Mental Health Status of First-Year College Students in China?

Our analysis established differences in overall status and various sub-dimensions of mental health among first-year college students in China. While the overall self-reported mental health of first-year college students was relatively good, they exhibited varying degrees of “somatic symptoms”. This suggests that despite their generally positive mental health, they may experience underlying issues related to physical health. This could be due to the various challenges they face as new students entering university, such as adapting to a new environment, academic pressure, and changes in their daily routine, which may lead to physical discomfort or illness (Yang et al., 2022). Previous studies have shown that there are not only significant direct effects between mental health and physical health, but also indirect effects mediated by physical activity (Ohrnberger et al., 2017). Therefore, to improve the mental health performance of first-year college students in terms of “somatic symptoms”, they can be encouraged to engage in appropriate physical exercise.

### 5.2. RQ2: How Does Mental Health Status Differ Among Student Demographics?

Gender, only-child status, and location of upbringing did not strongly influence the mental health scores of first-year college students in China. There are two possible explanations for these findings: First, the emphasis on equal education in China’s university system, coupled with relatively affordable tuition fees, has allowed many aspiring students to pursue higher education, thereby minimizing psychological issues arising from individual differences. Second, the rapid development of modern technology has facilitated the rapid dissemination and sharing of information. Indeed, cultural exchange and integration of urban and rural areas have continually expanded (Fang, 2022), potentially narrowing mental health disparities among students from diverse backgrounds. For example, the widespread access to the internet has made it easier for students to acquire knowledge about mental health and engage in online psychological counseling activities (Barak & Grohol, 2011).

### 5.3. RQ3: What Daily Behaviors Significantly Predict Student Mental Health Status?

The core finding of this study is the discovery of five daily behavioral factors that are significantly associated with the mental health of college students. Among them, sleep quality plays a particularly prominent role; nearly half of all interviewees mentioned its influence on their emotions. In summarizing the perspectives shared by the respondents, it can be concluded that poor sleep often makes it difficult to maintain a stable emotional state; this can lead to issues such as inattention and memory decline, which can then trigger feelings of irritation, anxiety, or depression. Conversely, good sleep can restore energy, sharpen the mind, and contribute to emotional stability. This is consistent with the conclusions of previous research (A. J. Scott et al., 2021). Moreover, our findings identified sleep quality as a key behavioral factor, while sleep duration was not selected in the final model. This result is consistent with the findings of Dagani et al. (2024).

In addition to sleep quality, physical exercise shows a notable positive association with mental health, effectively alleviating anxiety symptoms and significantly enhancing emotional states (Y. Li et al., 2021). Our study corroborates this finding, and further reveals that the frequency of “planned and implemented” exercise is the crucial factor that influences students’ mental health. However, it is important to acknowledge that higher levels of mental health may also promote better behaviors. Therefore, although the behavioral indicators examined in this study are predictive of mental health outcomes, we refrain from making definitive causal claims.

Intriguingly, among the numerous daily behaviors investigated, only sleep and physical exercise are unequivocally identified as key factors that influence mental health. In contrast, the three other variables—self-evaluation, daily average number of planned tasks, and task completion rate—are not specific behavioral categories, but rather indicators that reflect students’ locus of control during the process of recording their activities. These variables encompass individual abilities of planning, reflecting, and adjusting, namely, their level of self-management. Self-management describes a lifestyle which prioritizes self-organization and the ability to maintain self-determination over a healthy and long life (Zavydivska et al., 2016). Research has shown that good self-management skills can help college students cope with challenges and pressures in life, enhance their self-efficacy, and improve their mental health (Zimmerman, 1990). Our findings confirm this; it is not the behaviors themselves that significantly impact mental health, but rather the self-management demonstrated through those behaviors.

Surprisingly, social activities were not identified as a factor influencing mental health, contrary to the conclusions found in previous literature, which suggested that leisure time spent engaging in social activities had a significant impact on the mental health of college students (Byrd & McKinney, 2012; Das et al., 2019). The perceived impact of social activities was brought up in student interviews; several interviewees voluntarily mentioned the relationship between social activities and emotions, believing that interactions with friends, classmates, or family members would affect their emotional states. However, this belief was not substantiated by the quantitative results; we did not find ‘social activities’ to be a significant factor influencing mental health scores. One possible explanation is that some students may cope emotionally through solitude rather than frequent social activities. It is also possible that the complex emotional responses experienced in social settings (i.e., coexisting positive and negative feelings) could also influence the overall impact of social activities on mental health.

## 6. Conclusions

This study investigated the impact of daily behavioral factors on the mental health of first-year college students. Our results indicate that two specific behavioral variables (sleep quality, planned and implemented physical exercise) and three self-management variables (average of self-evaluation scores, average daily number of planned tasks, and the completion rate of planned tasks) are the key factors influencing the mental health of freshman students.

This study offers two unique contributions to the field of mental health research in college students: First, our research has confirmed the positive impacts of sleep quality, physical exercise, and self-management on mental health, and further elucidated the contributing factors involved. This discovery helps us gain a deeper understanding of the behaviors that affect the mental health of first-year college students from the perspective of their daily life. Second, departing from previous studies that focused on daily habits that affect mental health, we innovatively adopted a digital recording format and collected data through a mini program. This allowed participants to comprehensively record their behavioral data over a specific period. This technique enabled the discovery that not only the daily behaviors themselves but also the self-management of these behaviors is an important factor influencing mental health. This finding not only enriches relevant theoretical knowledge and provides a new research perspective for future studies, but also provides a new approach for investigating and improving the mental health of first-year college students.

### 6.1. Practical Implications

We propose the following implementations of these findings: First, college students should fully recognize the importance of sleep for physical and mental health, arrange their daily routine reasonably, and avoid unhealthy sleep habits such as staying up late. When college students experience excessive negative emotions or high stress, they can consider exercising to relax and improve their mood. It is advisable to plan and adhere to regular exercise to achieve the optimal effects on relaxation and mental health. Furthermore, college students should set clear plans and goals, strive to improve their completion rate, and learn to acknowledge their own strengths. This can be achieved through self-evaluating with a positive and objective attitude, thereby promoting the improvement of their mental health.

Second, we recommend that universities organize lectures on sleep health and prioritize the construction of dormitory environments to assist college students in improving their sleep quality. Additionally, updating sports facilities and offering more exercise courses may encourage students to actively engage in physical exercise and enjoy the benefits. To help students become better planners, universities can offer specialized self-management courses that teach practical skills to improve their planning and execution abilities, cope with the challenges of academia and daily life, and further promote their mental health.

### 6.2. Limitations and Future Research

We acknowledge that this study has certain limitations. First, the sample was relatively small due to the demands of long-term, continuous data collection, which required sustained commitment from participants. Additionally, all participants were first-year students from a single university in China, which limits the generalizability of the findings. Second, although interview data were collected, the qualitative component was relatively limited and served only as a supplementary method to support the quantitative findings. Third, this study primarily focused on identifying statistical predictors of mental health without examining potential moderating or mediating factors such as emotion regulation (Mastrokoukou et al., 2024). This represents an important direction for future research. Finally, despite a range of ethical safeguards—such as voluntary participation and anonymized data handling—minor observer effects or response bias may still have occurred due to the dual role of two authors as course instructors and participants’ awareness of being observed.

Although there may be some threats, the use of an innovative behavioral recording tool over a period of time, a well-validated mental health scale, and an appropriate analytical method suggests the results are trustworthy. Future studies could diversify the population and increase the sample size by involving universities from several regions, thus enhancing representation and generalizability of the findings. It is also important to improve our qualitative research methods by designing more in-depth and detailed interviews to obtain richer and more valuable information. Moreover, future research may benefit from more carefully designed studies that collect dynamic behavioral and mental health data over a longer period to support longitudinal modeling, which may provide deeper insights into the temporal mechanisms linking behavior and psychological well-being. These methods would help uncover the causal relationships and the long-term impact of specific behaviors on psychological well-being. In addition, future studies could explore potential mediators and moderators that influence college students’ mental health, further clarifying the pathways through which behaviors affect psychological outcomes.

## Figures and Tables

**Table 1:** Participant demographic characteristics.

Basic Information	Categories	Number	Percentage
Gender	Male	47	42.70%
	Female	63	57.30%
Place of upbringing	Provincial capital city	12	10.90%
	County-level city or prefecture-level city	50	45.50%
	Town	15	13.60%
	Rural area	33	30.00%
Only child	Yes	30	27.30%
	No	80	72.70%

**Table 2:** Reliability and validity indicators for each subscale.

Subscale	Cronbach’s Alpha	AVE	CR
somatic symptoms	0.81	0.45	0.83
anxiety and insomnia	0.91	0.59	0.91
social dysfunction	0.66	0.41	0.79
severe depression	0.94	0.70	0.94

**Table 3:** Measurement of daily behaviors.

Behavioral Category	Extracted Variables
Sleep	Average duration ^a^/quality ^b^
Classroom learning	Evaluation frequency ^c^
Reading	Planned and implemented ^c^/Planned but unimplemented ^c^/Unplanned but implemented ^c^/Unplanned and unimplemented ^c^/Frequency of recording ^c^/Average duration ^d^/Evaluation frequency ^c^
Physical exercise	Planned and implemented ^c^/Planned but unimplemented ^c^/Unplanned but implemented ^c^/Unplanned and unimplemented ^c^/Frequency of recording ^c^/Average duration ^d^/Evaluation frequency ^c^
Electronic products	Planned and implemented ^c^/Planned but unimplemented ^c^/Unplanned but implemented ^c^/Unplanned and unimplemented ^c^/Frequency of learning recording ^c^/Average duration of learning ^d^/Frequency of entertainment recording ^c^/Average duration of entertainment ^d^/Evaluation frequency ^c^
Hobbies and interests	Planned and implemented ^c^/Planned but unimplemented ^c^/Unplanned but implemented ^c^/Unplanned and unimplemented ^c^/Frequency of recording ^c^/Average duration ^d^/Evaluation frequency ^c^
Social activities	Planned and implemented ^c^/Planned but unimplemented ^c^/Unplanned but implemented ^c^/Unplanned and unimplemented ^c^/Frequency of recording ^c^/Average duration ^d^/Evaluation frequency ^c^
Overall situation	Frequency of recording self-evaluation scores ^c^/Average of self-evaluation scores ^e^/Today’s reflection ^c^/Days of recording ^c^/Average daily number of planned tasks ^c^/Average daily number of completed tasks ^c^/Completion rate of planned tasks ^f^

Note: ^a^ continuous variables range from 0 to 24 h of sleep; ^b^ ordinal variables range from 1 to 4, indicating sleep quality from poor to excellent; ^c^ discrete variables range from 0 to 28, measuring the frequency of behaviors; ^d^ continuous variables range from 0 to 1440 min; ^e^ ordinal variables range from 1 to 5, representing self-evaluation scores from negative to positive; ^f^ continuous variables range from 0 to 1, indicating the completion rate as a percentage.

**Table 4:** Distribution of responses on the mental health questionnaire.

Constructs	Items	Mean 1	Mean 2	SD
A scale “somatic symptoms”	7	24.95	3.56	4.82
B scale “anxiety and insomnia”	7	26.81	3.83	5.84
C scale “social dysfunction”	7	25.06	3.58	3.48
D scale “severe depression”	7	30.28	4.33	5.55
Total score of GHQ-28	28	107.10	3.83	15.48

Note: Mean 1 is the mean score of the total score for the dimension, while Mean 2 represents the average score per item.

**Table 5:** Results of one-way ANOVA.

Variables	Categories	Mean	SD	df	F	Sig.
Gender	Male	105.23	16.96	(1, 108)	1.195	0.277
	Female	108.49	14.26			
Only child	Yes	106.17	15.86	(1, 108)	0.149	0.700
	No	107.45	15.42			
Place of upbringing	Provincial capital city	112.25	10.02	(3, 106)	0.876	0.456
	County-level city or prefecture-level city	104.90	16.68			
	Town	109.13	11.28			
	Rural area	107.64	16.74			

**Table 6:** Relationship between influencing factors and mental health dimensions demonstrated through the LASSO regression model.

Influencing Factors	Mental Health				
	Overall Status	Somatic Symptoms	Anxiety and Insomnia	Social Dysfunction	Severe Depression
Sleep quality	β = 5.599	β = 0.466	β = 1.539	β = 0.597	β = 1.461
Planned and implemented physical exercise	β = 0.217	excl.	excl.	excl.	excl.
Evaluation frequency of physical exercise	excl.	excl.	excl.	excl.	β = 0.465
Frequency of recording self-evaluation scores	excl.	excl.	excl.	excl.	β = 0.465
Average of self-evaluation scores	β = 0.099	excl.	excl.	excl.	excl.
Average daily number of planned tasks	β = 0.972	excl.	excl.	excl.	β = 0.674
Completion rate of planned tasks	β = 1.637	excl.	excl.	β = 0.568	β = 0.117
Model determination coefficient	0.2794	0.0506	0.1595	0.1822	0.2470

Note: “excl.” stands for “The factors were excluded from the final Lasso results”.

## Data Availability

The data presented in this study are openly available in the Mendeley Data database at https://data.mendeley.com/datasets/jtmnv66mbg/1 (accessed on 7 January 2025).

## Supplementary Materials

The following supporting information can be downloaded at https://www.mdpi.com/article/10.3390/bs15050618/s1, Table S1: Mental health questionnaire items and percentage of each option; Table S2: Descriptive statistics and K–S test for daily behavioral variables (N = 110); Table S3: Tolerance and Variance Inflation Factor (VIF) values for daily behavioral variables; Table S4: Correlation matrix among daily behavioral variables; Table S5: Tolerance and Variance Inflation Factor (VIF) values for daily behavioral variables selected by LASSO regression.
