# Supplementary material for: Factors Influencing the Mental Health of First-Year College Students: Evidence from Digital Records of Daily Behaviors
Source: Behav Sci (Basel). 2025 May 2;15(5):618. doi: 10.3390/bs15050618 (PMC12109279; doi:10.3390/bs15050618)
Supplement: Supplementary file 1 [file behavsci-15-00618-s001.zip › Supplementary Table S1.pdf]

Supplementary Table S1. Mental health questionnaire items and percentage of each option.

Item statement	Strongly agree	Agree	Neutral	Disagree	Strongly disagree
**Somatic symptoms (Mean=3.56, SD=4.82)**					
Have you been feeling perfectly well and in good health? *	32 (29.1%)	54 (49.1%)	14 (12.7%)	10 (9.1%)	0
Have you been feeling in need of nutrients?	13 (11.8%)	53 (48.2%)	20 (18.2%)	22 (20.0%)	2 (1.8%)
Have you been feeling run down and out of sorts?	5 (4.5%)	24 (21.8%)	26 (23.6%)	43 (39.1%)	12 (10.9%)
Have you felt that you are ill?	2 (1.8%)	14 (12.7%)	18 (16.4%)	50 (45.5%)	26 (23.6%)
Have you been getting any pain in your head?	1 (0.9%)	19 (17.3%)	13 (11.8%)	50 (45.5%)	27 (24.5%)
Have you been getting a feeling of tightness or pressure in your head?	2 (1.8%)	20 (18.2%)	14 (12.7%)	50 (45.5%)	24 (21.8%)
Have you been having hot or cold spells?	1 (0.9%)	14 (12.7%)	9 (8.2%)	51 (46.4%)	35 (31.8%)
**Anxiety and insomnia (Mean=3.83, SD=5.84)**					
Have you lost much sleep over worry?	2 (1.8%)	16 (14.5%)	12 (10.9%)	51 (46.4%)	29 (26.4%)
Have you had difficulty in staying asleep once you fall asleep?	3 (2.7%)	13 (11.8%)	11 (10.0%)	44 (40.0%)	39 (35.5%)
Have you felt constantly under strain?	2 (1.8%)	16 (14.5%)	11 (10.0%)	52 (47.3%)	29 (26.4%)
Have you been getting edgy and bad-tempered?	3 (2.7%)	10 (9.1%)	11 (10.0%)	56 (50.9%)	30 (27.3%)
Have you been getting scared or panicky for no good reason?	2 (1.8%)	11 (10.0%)	7 (6.4%)	55 (50.0%)	35 (31.8%)
Do problems seem to be piling up for you?	3 (2.7%)	20 (18.2%)	22 (20.0%)	43 (39.1%)	22 (20.0%)

Have you been feeling nervous and strung up all the time?	3 (2.7%)	14 (12.7%)	14 (12.7%)	52 (47.3%)	27 (24.5%)
**Social dysfunction (Mean=3.58, SD=3.48)**					
Have you been managing to keep yourself busy and occupied? *	7 (6.4%)	33 (30.0%)	29 (26.4%)	27 (24.5%)	14 (12.7%)
Have you been taking longer to accomplish things?	3 (2.7%)	30 (27.3%)	23 (20.9%)	38 (34.5%)	16 (14.5%)
Have you felt on the whole you were doing things well? *	8 (7.3%)	63 (57.3%)	26 (23.6%)	12 (10.9%)	1 (0.9%)
Have you been satisfied with the way you've carried out your task? *	9 (8.2%)	52 (47.3%)	32 (29.1%)	15 (13.6%)	2 (1.8%)
Have you felt that you are playing a useful part in things? *	11 (10.0%)	80 (72.7%)	15 (13.6%)	3 (2.7%)	1 (0.9%)
Have you felt capable of making decisions about things? *	14 (12.7%)	78 (70.9%)	14 (12.7%)	4 (3.6%)	0
Have you been able to enjoy your normal day-to-day activities? *	19 (17.3%)	72 (65.5%)	15 (13.6%)	4 (3.6%)	0
**Severe depression (Mean=4.33, SD=5.55)**					
Have you been thinking of yourself as a worthless person?	1 (0.9%)	4 (3.6%)	11 (10.0%)	35 (31.8%)	59 (53.6%)
Have you felt that life is entirely hopeless?	0	4 (3.6%)	11 (10.0%)	29 (26.4%)	66 (60.0%)
Have you felt that life isn't worth living?	0	7 (6.4%)	10 (9.1%)	27 (24.5%)	66 (60.0%)
Have you had thoughts of committing suicide?	2 (1.8%)	7 (6.4%)	10 (9.1%)	28 (25.5%)	63 (57.3%)
Have you found at times you couldn't do anything because your nerves were too bad?	0	14 (12.7%)	19 (17.3%)	25 (22.7%)	52 (47.3%)
Have you found yourself wishing you were dead and away from it all?	1 (0.9%)	9 (8.2%)	11 (10.0%)	24 (21.8%)	65 (59.1%)
Do you seem to have persistent thoughts about taking your own life?	0	4 (3.6%)	8 (7.3%)	28 (25.5%)	70 (63.6%)

Note: Items marked with an asterisk (*) are reverse items, which means that a higher score indicates a poorer mental health status.
