# Supplementary material for: Factors Influencing the Mental Health of First-Year College Students: Evidence from Digital Records of Daily Behaviors
Source: Behav Sci (Basel). 2025 May 2;15(5):618. doi: 10.3390/bs15050618 (PMC12109279; doi:10.3390/bs15050618)
Supplement: Supplementary file 1 [file behavsci-15-00618-s001.zip › Supplementary Table S2.pdf]

Supplementary Table S2. Descriptive statistics and K–S test for daily behavioral variables (N=110).

**Daily behavioral variables**	**Mean**	**SD**	**Sig.(K-S)**
Sleep duration	7.687	0.903	0.000
Sleep quality	3.250	0.608	0.000
Classroom learning—evaluation frequency	23.430	5.916	0.000
Reading—planned and implemented	5.870	7.840	0.000
Reading—planned but unimplemented	0.540	2.395	0.000
Reading—unplanned but implemented	0.080	0.491	0.000
Reading—unplanned and unimplemented	19.420	8.520	0.000
Reading—recording frequency	5.250	7.596	0.000
Reading—duration	34.659	45.215	0.000
Reading—evaluation frequency	5.750	7.765	0.000
Physical exercise—planned and implemented	3.950	5.539	0.000
Physical exercise—planned but unimplemented	0.100	0.357	0.000
Physical exercise—unplanned but implemented	0.310	2.587	0.000
Physical exercise—unplanned and unimplemented	21.550	7.085	0.000
Physical exercise—recording frequency	3.890	5.760	0.000
Physical exercise—duration	38.049	50.861	0.000
Physical exercise—evaluation frequency	4.460	6.762	0.000
Electronic products—planned and implemented	8.350	10.094	0.000
Electronic products—planned but unimplemented	0.180	0.859	0.000
Electronic products—unplanned but implemented	0.550	3.204	0.000
Electronic products—unplanned and unimplemented	16.840	10.703	0.000
Electronic products—frequency of learning recording	7.620	9.470	0.000
Electronic products—duration of learning	58.617	74.676	0.000

Electronic products—frequency of entertainment recording	8.150	10.069	0.000
Electronic products—duration of entertainment	84.962	101.661	0.000
Electronic products—evaluation frequency	8.280	9.854	0.000
Hobbies and interests—planned and implemented	3.010	5.091	0.000
Hobbies and interests—planned but unimplemented	0.250	1.344	0.000
Hobbies and interests—unplanned but implemented	0.030	0.212	0.000
Hobbies and interests—unplanned and unimplemented	22.610	6.440	0.000
Hobbies and interests—recording frequency	2.730	4.874	0.000
Hobbies and interests—duration	35.531	54.738	0.000
Hobbies and interests—evaluation frequency	3.050	5.110	0.000
Social activities—planned and implemented	3.260	4.836	0.000
Social activities—planned but unimplemented	0.250	1.068	0.000
Social activities—unplanned but implemented	0.130	0.858	0.000
Social activities—unplanned and unimplemented	22.260	6.200	0.000
Social activities—recording frequency	3.200	4.615	0.000
Social activities—duration	58.969	89.508	0.000
Social activities—evaluation frequency	3.350	4.847	0.000
Frequency of recording self-evaluation scores	23.180	7.675	0.000
Average of self-evaluation scores	4.225	3.895	0.000
Today's reflection	3.900	7.434	0.000
Days of recording	25.920	4.548	0.000
Average daily number of planned tasks	2.344	1.852	0.000
Average daily number of completed tasks	2.395	1.842	0.000
Completion rate of planned tasks	0.611	0.323	0.000
