# Supplementary material for: Factors Influencing the Mental Health of First-Year College Students: Evidence from Digital Records of Daily Behaviors
Source: Behav Sci (Basel). 2025 May 2;15(5):618. doi: 10.3390/bs15050618 (PMC12109279; doi:10.3390/bs15050618)
Supplement: Supplementary file 1 [file behavsci-15-00618-s001.zip › Supplementary Table S3.pdf]

Supplementary Table S3. Tolerance and Variance Inflation Factor (VIF) values for daily behavioral variables.

**Daily behavioral variables**	**Tolerance**	**VIF**
Sleep duration	0.544	1.838
Sleep quality	0.545	1.836
Classroom learning—evaluation frequency	0.215	4.646
Reading—planned and implemented	0.001	1218.156
Reading—planned but unimplemented	0.008	124.905
Reading—unplanned but implemented	0.048	20.681
Reading—unplanned and unimplemented	0.001	1370.287
Reading—recording frequency	0.062	16.089
Reading—duration	0.377	2.654
Reading—evaluation frequency	0.024	41.763
Physical exercise—planned and implemented	0.001	1304.741
Physical exercise—planned but unimplemented	0.116	8.630
Physical exercise—unplanned but implemented	0.004	279.928
Physical exercise—unplanned and unimplemented	0.001	1990.110
Physical exercise—recording frequency	0.012	85.904
Physical exercise—duration	0.404	2.477
Physical exercise—evaluation frequency	0.151	6.616
Electronic products—planned and implemented	0.036	28.116
Electronic products—planned but unimplemented	0.070	14.260
Electronic products—unplanned but implemented	0.010	103.947
Electronic products—unplanned and unimplemented	0.038	26.058
Electronic products—frequency of learning recording	0.232	4.318
Electronic products—duration of learning	0.017	59.843

Electronic products—frequency of entertainment recording	0.241	4.152
Electronic products—duration of entertainment	0.017	59.544
Electronic products—evaluation frequency	0.001	1057.576
Hobbies and interests—planned and implemented	0.070	14.281
Hobbies and interests—planned but unimplemented	0.006	170.308
Hobbies and interests—unplanned but implemented	0.009	114.098
Hobbies and interests—unplanned and unimplemented	0.329	3.039
Hobbies and interests—recording frequency	0.001	1209.204
Hobbies and interests—duration	0.001	866.255
Hobbies and interests—evaluation frequency	0.020	49.510
Social activities—planned and implemented	0.011	91.433
Social activities—planned but unimplemented	0.001	969.502
Social activities—unplanned but implemented	0.007	143.151
Social activities—unplanned and unimplemented	0.311	3.211
Social activities—recording frequency	0.004	238.629
Social activities—duration	0.361	2.773
Social activities—evaluation frequency	0.567	1.764
Frequency of recording self-evaluation scores	0.324	3.086
Average of self-evaluation scores	0.002	608.880
Today's reflection	0.002	405.446
Days of recording	0.003	337.562
Average daily number of planned tasks	0.242	4.136
Average daily number of completed tasks	0.544	1.838
Completion rate of planned tasks	0.545	1.836
