# Supplementary material for: Factors Influencing the Mental Health of First-Year College Students: Evidence from Digital Records of Daily Behaviors
Source: Behav Sci (Basel). 2025 May 2;15(5):618. doi: 10.3390/bs15050618 (PMC12109279; doi:10.3390/bs15050618)
Supplement: Supplementary file 1 [file behavsci-15-00618-s001.zip › Supplementary Table S5.pdf]

Supplementary Table S5. Tolerance and Variance Inflation Factor (VIF) values for daily behavioral variables selected by LASSO regression.

**Daily behavioral variables selected by LASSO regression**	**Tolerance**	**VIF**
Sleep quality	0.963	1.038
Physical exercise—planned and implemented	0.515	1.943
Average of self-evaluation scores	0.984	1.016
Average daily number of planned tasks	0.361	2.773
Completion rate of planned tasks	0.554	1.804
